# The Stomatoscope

**Published:** 1881-08

**Authors:** 


*The Stomatoscope.*—Dr. S. G. Robertson, of Eufaula, Ala., sends the following description of this instrument:

Dr. Bodecker, of New York, introduced in this country the Grohnwald Stomatoscope by having one sent over from Berlin. His attention was called to the merits of this light in excavating and filling teeth, by Dr. Turnofsky, Budopost. I corresponded with Dr. Bodecker on the subject of getting one, and after some delay and disappointment, he succeeded in having six put up in this country, to order, at a cost of $100 each. Since giving it a thorough trial, under severe tests, with olar lights, I had rather go back to old principles than be without this light. All difficulties in getting a perfect view of distal and difficult cavities are now removed—even the darkest night, without the aid of any additional light. When the day is cloudy I darken my operating room and operate under it altogether. The full confidence in my own efforts to do perfect work under this light has been well tested by comparison with my efforts under the best daylight—not that artificial light is better than solar light, only in the concentration of the rays upon the tooth to be operated on. It must be seen to be appreciated. The difference in a good view with the Stomatoscope and incident solar light, will approximate the difference between objects seen under a microscope and the eye unaided. It is quite simple when understood. It consists of a 4x8 inch tube, with a light in the center, a reflector in the posterior and a plano convex lens in the anterior end. The rays are collected upon a concave mirror and thrown into the mouth at a focal distance of ten to fourteen inches.

The first week I made half the value of the instrument that would not have otherwise been made. All the heat felt at the mouth is the actinic rays, which is scarcely perceptible to the patient.

I have been requested by other journals to give them an account of the light, which I have done, or else should go into particulars now, but as Dentists will see it in other places, it is not necessary to do more than call their attention to it here.

